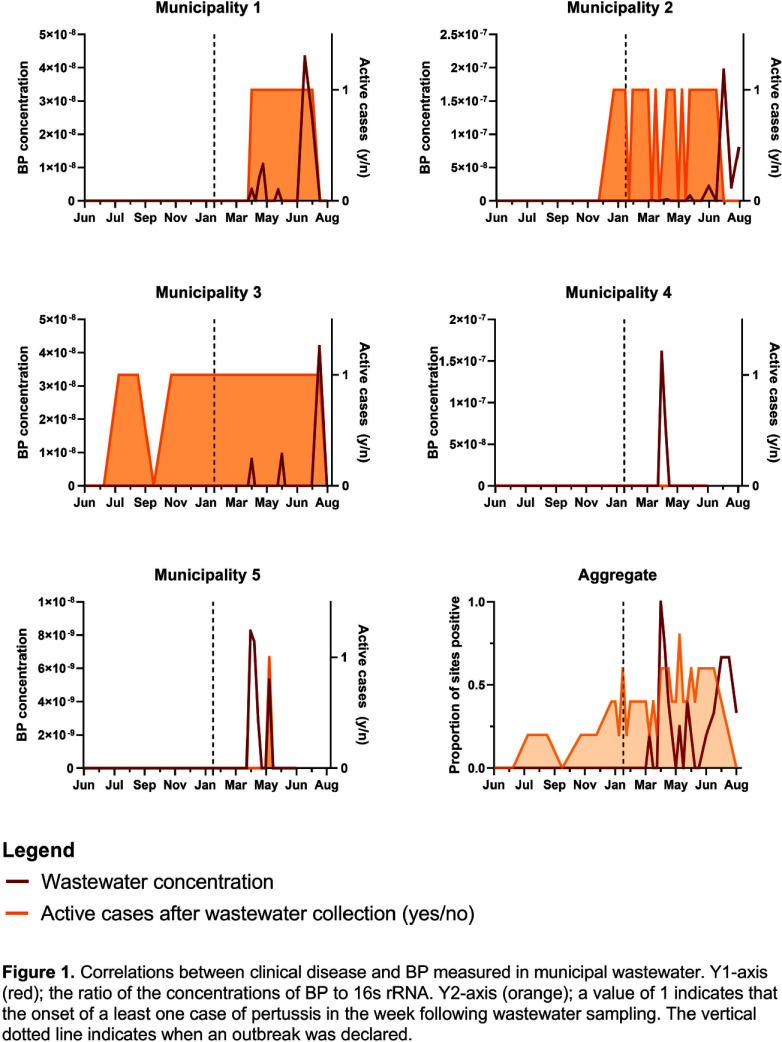# Wastewater-based surveillance of Bordetella pertussis during an outbreak in Alberta, Canada

**DOI:** 10.1017/ash.2025.388

**Published:** 2025-09-24

**Authors:** R. Benson Weyant, Catalina Gonzalez, Barbara Wadell, Kristine Du, Jangwoo Lee, Nicole Acosta, Maria Chavarriaga, Rhonda Clark, Mila Luchak, Jordan Ross, Xiaoli Pang, Bonita Lee, Christine O’Grady, Casey Hubert, Michael Parkins

**Affiliations:** 1 University of Calgary; 2 University of Alberta

## Abstract

**Background:** Whooping cough, caused by Bordetella pertussis (BP), is a vaccine-preventable illness spread through respiratory droplets. As the disease disproportionally impacts infants and children, vaccination is part of the routine childhood series in Canada. However, vaccine hesitancy and resultant declining rates of community immunity increase the risk of disease. We leveraged our existing wastewater-based surveillance (WBS) network to assess its ability to track clinical disease in response to an outbreak in southern Alberta. **Methods:** For seven months before and after the declaration of a January 2023 outbreak, wastewater samples were collected at approximately weekly intervals from five municipalities in southern Alberta (~1.05 million residents). 24-hour composite wastewater was pelleted, mechanically lysed, and DNA extracted. B. pertussis gene BP283 was quantified by qPCR and normalized against total-bacterial 16s rRNA. De-identified clinical data was obtained from Alberta Health Services (AHS) and vaccination rates collected from the AHS Interactive Health Data Application dashboard (http://www.ahw.gov.ab.ca/IHDA_Retrieval/). Per local guidelines, cases could be diagnosed through molecular testing, or if an individual had a strong epidemiological link to a known case and compatible whooping cough symptoms (https://open.alberta.ca/publications/pertussis). Cases were mapped to sewershed areas using forward sortation areas. Fishers exact test was used to determine the association of the categorical variables of positive wastewater samples and clinical cases diagnosed in the week following, as well as to compare rates of wastewater positivity before and after the outbreak was declared. **Results:** Over the study period 296 cases of whooping cough were identified, with 256 after the outbreak was declared. No wastewater samples were positive for BP prior to the outbreak (0%) and 22 were positive during the outbreak (19.8%), p = 0.0006 (Figure 1). Of the positives, the median ratio of BP:16s rRNA was 8.89x10-9 (IQR: 5.31x10-9 to 4.19x10-8). Detection of BP in the wastewater did not necessarily predict the occurrence of cases the following week within individual municipalities, but there was an association when all sites were aggregated (OR 2.47 [CI: 1.01–6.05], p = 0.04). Vaccination rates in the communities ranged from 40.9% to 72.2%, and did not associate with wastewater detected Bordetella pertussis. **Conclusion:** BP can be detected in wastewater during outbreak periods, though infrequently and in very low concentrations. For WBS to be used as an effective tool to monitor and potentially mitigate cases of whooping cough for WBS (and other respiratory pathogens which are not readily amplified in the gastrointestinal tract), assay sensitivity will need to be improved.

**Figure:**